# Male diet affects female fitness and sperm competition in human- and bat-associated lineages of the common bedbug, *Cimex lectularius*

**DOI:** 10.1038/s41598-021-94622-6

**Published:** 2021-07-30

**Authors:** Jana Křemenová, Tomáš Bartonička, Ondřej Balvín, Christian Massino, Klaus Reinhardt, Markéta Sasínková, Alfons R. Weig, Oliver Otti

**Affiliations:** 1 Department of Botany and Zoology, Faculty of Science, Masaryk University, Kotlářská 2, 611 37 Brno, Czech Republic; 2 Department of Ecology, Faculty of Environmental Sciences, Czech University of Life Sciences Prague, Kamýcká 129, 165 21 Prague 6, Czech Republic; 3 Applied Zoology, Department of Biology, Technische Universität Dresden, 01062 Dresden, Germany; 4 Genomics and Bioinformatics, Bayreuth Center of Ecology and Environmental Research (BayCEER), University of Bayreuth, Universitätsstrasse 30, 95440 Bayreuth, Germany; 5 Animal Population Ecology, Animal Ecology I, University of Bayreuth, Universitätsstrasse 30, 95440 Bayreuth, Germany

**Keywords:** Evolutionary ecology, Speciation, Entomology, Evolutionary ecology

## Abstract

Sperm performance can vary in ecologically divergent populations, but it is often not clear whether the environment per se or genomic differences arising from divergent selection cause the difference. One powerful and easily manipulated environmental effect is diet. Populations of bedbugs (*Cimex lectularius*) naturally feed either on bat or human blood. These are diverging genetically into a bat-associated and a human-associated lineage. To measure how male diet affects sperm performance, we kept males of two HL and BL populations each on either their own or the foreign diet. Then we investigated male reproductive success in a single mating and sperm competition context. We found that male diet affected female fecundity and changed the outcome of sperm competition, at least in the human lineage. However, this influence of diet on sperm performance was moulded by an interaction. Bat blood generally had a beneficial effect on sperm competitiveness and seemed to be a better food source in both lineages. Few studies have examined the effects of male diet on sperm performance generally, and sperm competition specifically. Our results reinforce the importance to consider the environment in which sperm are produced. In the absence of gene flow, such differences may increase reproductive isolation. In the presence of gene flow, however, the generally better sperm performance after consuming bat blood suggests that the diet is likely to homogenise rather than isolate populations.

## Introduction

Speciation proceeds if reproductive isolation is based on genetic differences^1,2^. A key question in speciation is whether the reproductive barrier arises first, then reduces gene flow, and so drives populations and their ecological traits apart, i.e. reproductive isolation is a by-product of divergence^1^ or whether divergent natural selection on specific traits exists that act as direct reproductive barriers (ecological speciation^2^).

The key male fitness parameter is sperm performance, in either isolation or in competition. This significance is reflected by the existence of hundreds of studies on sperm competition^3–5^ and likely thousands of medical studies examining sperm (dys)function. For theoretical and diagnostic reasons, a central focus of these studies was fertility, or fitness, under predictions based on the male genotype. However, the male genotype is a relatively poor predictor of male's fertility^6^ and of sperm competition ability^7^, and it has a minute heritability^8^. Medical and evolutionary studies agree that environmental or lifestyle factors are responsible for a large part of the variation observed in sperm performance^7,9^.

Environmental factors can impact sperm performance in a number of ways, for example immediately or after a delay, they can act short-term or be long-lasting, and their impact may differ for different aspects of sperm performance. Many studies show variation in sperm performance in response to pH, substrate fluidity, temperature, sexually transmitted diseases, and many other environmental factors (e.g. Alavi and Cosson 2005^10^; Foresta et al. 2010^11^; Mann 1964^12^; Otti et al. 2013^13^; Valdebenito et al. 2009^14^; Werner and Simmons 2008^15^). In some cases, the environment causes phenotypic variation in sperm performance, such as changes in sperm motility due to temperature, altitude, or season in birds and mammals or changes in sperm defence ability due to nutrition in flour beetles (e.g. Barros et al. 2006^16^; Blanco et al. 2000^17^; Chacur et al. 2013^18^; Lewis et al. 2012^19^; Schramm 2008^20^; for more examples see also Table 1 in Reinhardt et al. 2015^7^). However, an important aspect of sperm performance is its longitudinal change, i.e. along the lifetime of sperm cell^7^. Sperm performance can evolve in response to the environment or respond plastically. This distinction is important for evolutionary research because if sperm performance is plastic, gene flow between ecologically separated populations is not hampered. Nevertheless, if sperm performance adapts locally, reproductive isolation may occur, such as that observed in male fruit fly fertility associated with temperature^21^ or different sperm motility in the substrate in mouthbrooding cichlid fish^22^. Given the overwhelming impact of environmental factors on sperm performance, there is a surprising paucity of studies addressing how environmental factors affect the main male fitness trait, sperm competition ability.

Diet represents a particularly important environmental factor for sperm performance for two main reasons, apart from immediate toxicity. First, lipids form the building block of cellular membranes, including sperm membranes^23,24^. Diet enriched by lipids, such as sterols and fatty acids, influences spermatogenesis, sperm viability, or male fertility^25–27^. Second, diet influences sperm energy metabolism^23,28–32^. For example, Ferramosca and Zara (2014)^33^ and Paynter et al. (2017)^30^ showed that sugars play an important role in sperm metabolic activity, potentially influencing sperm capacitation and viability. Similarly, a protein-deficient diet can reduce sperm motility, count and viability, fertility rate, sperm mitochondrial activity, or hyaluronidase activity^31,32,34^.

Moreover, the diet also influences the chemical composition of male seminal fluid^35,36^. Seminal fluid proteins induce numerous physiological and behavioural post-mating changes in female insects, i.e. affecting sperm storage parameters, increasing egg production, and modulating sperm competition^37,38^. As stated above, sperm performance may respond plastically or in a more fixed manner, but not all populations may respond in the same way to the same environment. Few data exist as to how different populations, or genotypes, respond to changes in the environment by differences in sperm performance, and fewer still separate the relative contribution of the environment and genotype^21,39,40^. The insufficient knowledge of environmental factors on sperm performance including sperm competition severely hampers our ability to predict evolutionary change, reproductive isolation, and ecological impacts.

Here we use a unique system to start addressing some of these issues. Populations of the common bedbug (*Cimex lectularius*) naturally live either on bats or humans where they consume the blood of their hosts. These bat- and human-associated populations fall into two genetic clades, which we call bat lineage (BL) and human lineage (HL)^41,42^. However, both clades can be reared on the blood of the other host, allowing us to separate environmental and genetic factors experimentally, and to examine for each clade the effects of own and foreign diet. Moreover, we used two populations from each BL and HL, allowing us to assign more confidently any effects on sperm to diet per se, to selection on bat or human blood (i.e., the BL or HL origin). Using one laboratory tester female population that is different from male populations, we reduced the variation due to females and measured various male fertility traits. Because diet might affect different aspects of sperm performance, we measured, in addition to male competitive fertilisation success, the number and proportion of fertilised eggs induced in tester females, and the duration of female fertility (reproductive senescence) after a single mating. By this way, we revealed one of the first examples of environmental effects on sperm competitive ability.

## Material and methods

### Bedbug culture and origin of populations

All bedbugs were maintained in an incubator at 26 ± 1 °C, at 70% relative humidity with a cycle of 12L:12D. All individuals in our study were virgin prior to the experiment. Bedbugs were fed weekly on bat and human blood (conserved by CPDA anticoagulant preservative solution; obtained from Faculty Hospital Bohunice, Brno with permission to use it for research purposes) using the protocol of Wawrocka and Bartonička (2013)^43^for bat blood and Aak and Rukke (2014)^44^ for human blood. Greater mouse-eared bats (*Myotis myotis*) were fed ad libitum with a mixed diet consisting of crickets (*Acheta spp.*) and mealworms (*Tenebrio molitor*) and after experiments, returned to the colony. All the protocols are carried out according to relevant guidelines and regulations. Experimental procedures were approved by the Ethical Committee of the Masaryk University (No. 1/2018). Bats were captured, handled, and temporarily kept in captivity under the licence issued by the South Moravian Regional Authority (Permit JMK 63761/2017). TB is authorised to handle free-living bats under the Certificate of Competency No. CZ01297 (§17, law No 246/1992), No. 922/93-OOP/2884/93 and 137/06/38/MK/E/07 of the Ministry of Environment of the Czech Republic.

The protocols of maintenance and generation of virgin individuals follow Reinhardt et al. (2003)^45^. We used individuals from five stock populations—three HL collected from human infestations and two BL populations from bat nursery colonies of greater mouse-eared bats (*Myotis myotis*) roosted in attics of churches. Populations were maintained in the laboratory for different amounts of time. In our experience, wild bedbug populations (bat and human) were habituated to laboratory conditions after 3 generations. Therefore, we do not expect variation caused by different times of laboratory rearing. We chose our oldest HL population collected in 2006 in London (in the laboratory for approximately 44 generations) to serve as a tester population for all experiments described in the following. The two other HL populations were collected in 2010, i.e. HL1 in Budapest and HL2 in Nairobi, and maintained in the laboratory at Universities of Sheffield and Bayreuth for approximately 36 generations. To ensure identical rearing conditions, all HL populations were transferred to Masaryk University in Brno in January 2018. BL populations were collected in Hanušovice (BL1) and Raškov (BL2) in 2016 and maintained in the laboratory at Masaryk University for 10 generations. All populations were reared on foreign blood for 3 generations before the start of experiment.

### Egg-laying rates and fertile period

We used virgin males from BL1, BL2, HL1, and HL2 reared on either their original or foreign diet (N_total_ = 92 virgin males, Supplementary Table S1) and measured their ability to induce egg-laying in tester females fed on human blood (their original diet). At fourteen days of age, every male was mated once with a fourteen-day old virgin female. Matings were staged, monitored, and interrupted after 60 s as described earlier^46^. Interrupted matings reduce variation in sperm number because of the linear relationship between copulation duration and sperm number^47^. A standardised sperm number was desirable since spermatozoa trigger the release of an oviposition-stimulating hormone from the *corpora allata*^48^ and could potentially influence lifespan through differential egg production. The use of 60 s standard mating also allows comparability with other studies^13,45,49–51^. After mating, the females were kept individually and sexually isolated in 15 mL plastic tubes equipped with a piece of filter paper for egg laying. Females were fed weekly, and the number of fertilised and unfertilised eggs was counted at weekly intervals for 10 weeks when all became infertile. The onset of infertility and fertilization senescence, the time point when the number of unfertilised eggs increased above 50% of the weekly clutch for the first time (similar to Reinhardt et al. 2009^50^), was used to determine the fertile duration each male was able to induce.

### Sperm competitive ability

Focal males from the previous experiment were also used to assess the effect of male diet on competitive fertilisation success and to perform paternity analyses (see DNA extraction, PCR, and fragment analysis with microsatellite markers). After the first mating, they were fed another two times a week apart and then mated with a new fourteen-day old tester female for 60 s that had previously been mated for 60 s with a tester virgin male of the same age (N_total_ = 79 virgin females and 79 males from F4 fed on human blood; see Supplementary Table S1). After mating, the females were kept individually and sexually isolated in 15 mL plastic tubes equipped with a piece of filter paper for egg laying. Females were fed weekly, and the number of fertilised and unfertilised eggs was counted at weekly intervals for 12 weeks when all became infertile. Eggs were stored and after hatching, nymphs were reared to the 3rd instar to have enough DNA for paternity analysis.

### DNA extraction, PCR, and paternity analysis using microsatellite markers

We extracted the DNA of the mothers, potential fathers and their offspring using the extraction chemicals of the PCRBioRapid kit (PB10.24, PCRBiosystems, London, UK). We randomly selected ten 3rd instar nymphs from each week of offspring production per cross (N = 22–112). For 62 mother-week combinations (12.5%) we had less than 10 offspring and in this case, we analysed all offspring. We used two different microsatellite primer mixes in multiplex PCRs (Supplementary Table S2 and S3) using the multiplex PCR kit from Qiagen (206,143, Qiagen, Hilden, Germany). In total, we used six microsatellite primer pairs from an earlier study by Fountain et al. (2014)^52^. The fragment analysis was done at the Genomics and Bioinformatics core facility at the University of Bayreuth using the Fragment Analyzer 5200 (Agilent Technologies, Waldbronn, Germany). Alleles were scored using the PROSize software (version 3.0, Agilent Technologies, Waldbronn, Germany). From the original 79 crosses (4589 offspring), we were able to successfully assign a father to 3921 offspring from 69 crosses.

### Statistical analysis

All statistical analyses were performed using R 4.0.3^53^ using the packages *lme4*^54^, *lmerTest*^55^, *glmmTMB*^56^, and *coxme*^57^.

#### Analysis of the induction of egg-laying and fertile period

##### Fertilised eggs

To analyse the effect of male diet on egg production, we fitted a linear mixed model (LME) with total number of fertile eggs as a response variable and male lineage (bat-/human-associated), diet (foreign/original) and their interaction terms as fixed factors and male population as a random effect. We checked for normality and homogeneity by visually inspecting residual versus fitted plots, the *qqnorm* function and Shapiro–Wilk test.

##### Proportion of fertilised eggs

We used the *cbind* function in R to combine unfertililised and fertilised eggs as a response variable. With this response variable, we then fitted a generalised linear mixed-effects model (GLME) with male lineage (bat-/human-associated) and diet (foreign/original) as fixed factors and male population as a random effect.

##### Onset of infertility

The package *coxme*^57^ was used to conduct survival analyses on the probability of laying unfertilised eggs and sperm performance loss. We fitted a cox proportional hazard model with male population as a random effect to investigate the fixed effects of male lineage, diet, and their interaction terms on fertilization senescence.

#### Analysis of sperm competitive ability

We investigated how the paternity of the focal male (P2) changes over time. Therefore, we used the *cbind* function to construct a response variable with the paternity of the focal male and the paternity of the tester male per week. Then we fitted GLME with lineage and diet of focal male, week and their 2-way interactions as fixed factors and with focal male population and family (combination of female, P1 and P2 males) as random effects.

To analyse total P2, we used the *cbind* function to combine the overall paternity of the focal male and of the tester male. For this response variable, we fitted GLME with above-mentioned fixed and random factors except “Week” and “Family”.

Because all our GLME models were overdispersed, we fitted all GLMEs with betabinomial distribution^58^ using the package *glmmTMB* (Brooks et al. 2017)^56^.

## Results

### Egg laying rate and fertile period

#### Fertilised eggs

**Figure 1:**
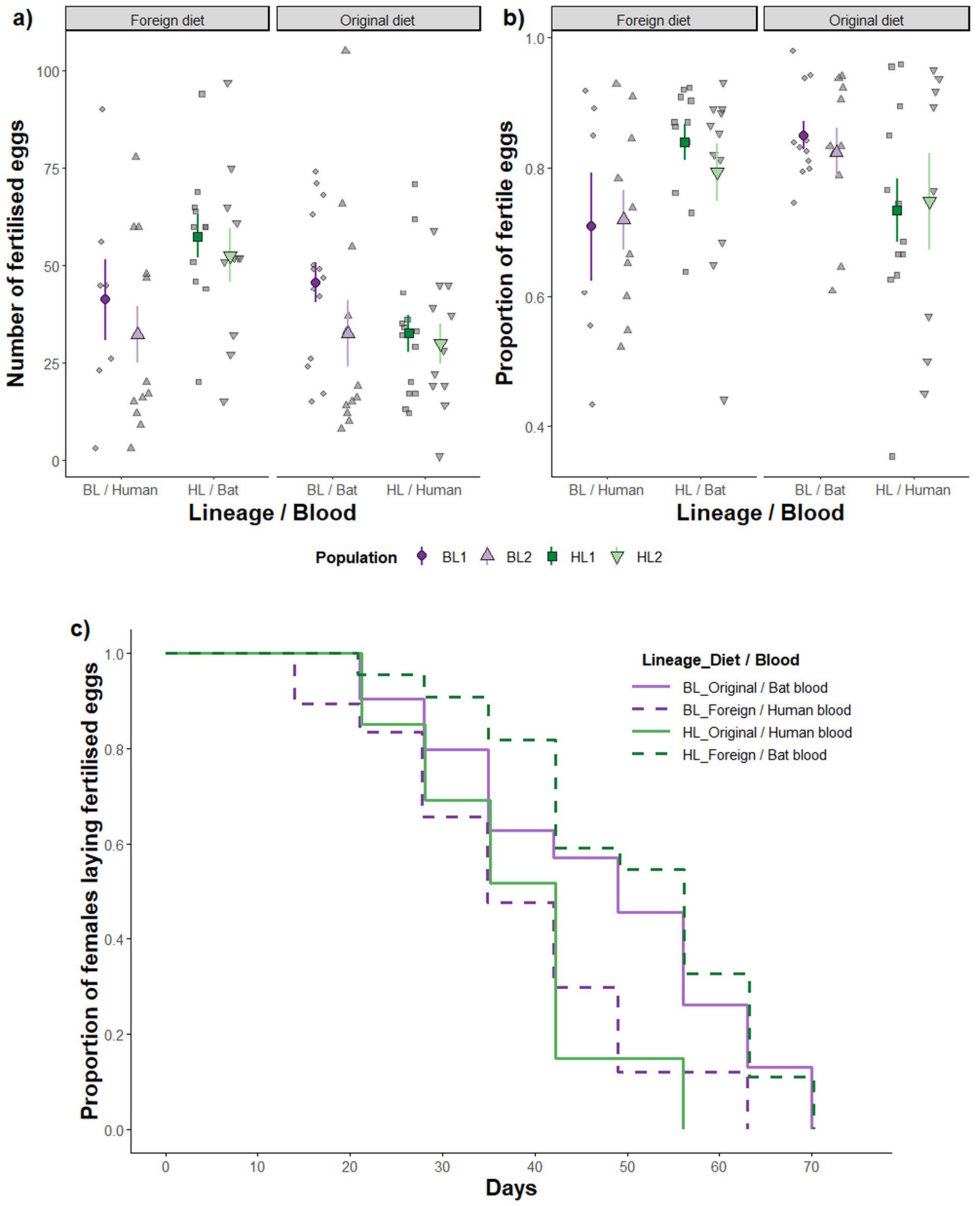
Effect of male diet (original or foreign, resp. bat or human blood) on male fertilisation success after a single mating with tester female in two different bedbug lineages (bat (BL, purple colour) or human (HL, green colour)). (**a**) The number of fertilised eggs and (**b**) the proportion of fertilised eggs (after exclusion of females laying only fertilised eggs) depended on the interaction of male diet and genetic origin. Different colour shades and point shapes with error bars show the mean and standard error for the individual male population. Grey symbols represent individual data points. (**c**)Induction of female infertility (more than 50% unfertilised eggs/clutch) changed with respect to male diet (solid lines correspond to the original diet, dashed lines to foreign diet) and lineage.

The number of fertilised eggs laid by females after a single mating showed a significant lineage:diet interaction effect (Table 1a). HL males induced more fertilised eggs when they are reared on a foreign diet than on their original diet, while BL males induced similar numbers of fertilised eggs on either diet (Table 1a, Fig. 1a). Fertilised egg numbers did not differ between male lineages (Table 1a, Fig. 1a) suggesting that the significant diet-induced effect is driven by the effect of bat blood on HL males.

**Table 1:** Results of statistical analysis of male diet (foreign/original) and lineage (bat/human) effects on fertilisation success after single mating with tester female (n = 92 couples). Values in bold represent significant effects and nearly significant effects are underlined.

(a) Number of fertilised eggs (LME)				
	F	*p*	Post-hoc tests	
			t	*p*
Lineage	0.84	0.459	2.44	0.053
Diet	4.92	**0.029**	0.51	0.610
Lineage : diet	8.73	**0.004**	−2.96	**0.004**

(b) Proportion of fertilised eggs (GLME)				
	χ^2^	*p*	Post-hoc tests	
			z	*p*
**All females**				
Lineage	0.00	0.990	–	–
Diet	3.66	0.056	–	–
**Only females laying one or more unfertilised eggs (n = 79 couples)**				
Lineage	3.23	0.072	−1.80	0.072
Diet	4.33	**0.038**	−2.08	**0.038**
Lineage : Diet	5.54	**0.019**	2.35	**0.019**

(c) Senescence (Wald test)				
	χ^2^	*p*	Post-hoc tests	
			z	*p*
Lineage	0.24	0.628	−2.07	**0.038**
Diet	0.15	0.697	−2.32	**0.020**
Lineage : Diet	11.25	** < 0.001**	3.35	** < 0.001**

#### Proportion of fertilised eggs

When we included all females into the model, the effect of male diet on the proportion of fertilised eggs was near to statistical significance (Table 1b). Lineage:diet interaction became significant (Table 1b) when females laying only fertilised eggs (less than 14%) were excluded. Males, regardless lineage, fed on original diet induced a smaller proportion of infertile eggs compared to foreign diet (Fig. 1b).

#### Onset of infertility

The time when males induced female infertility significantly depended on the interaction of lineage and diet (Table 1c). BLs fed on original diet and HLs on foreign diet (on bat blood) induced longer fertile periods than males on reverse diet (human blood), i.e. one week longer for BL males and two weeks longer for HL males (Fig. 1c).

### Sperm competitive ability

**Figure 2:**
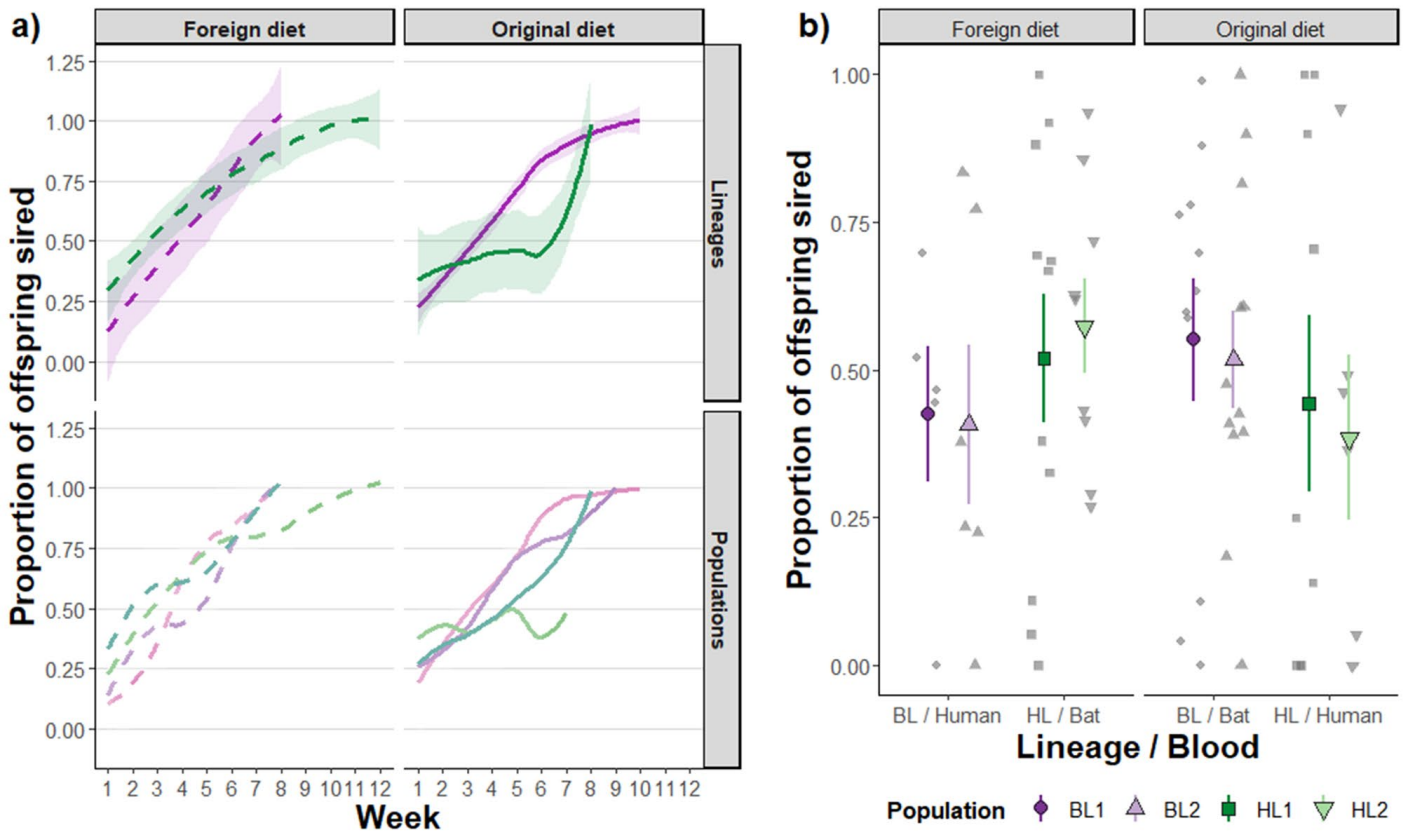
Effect of male diet on sperm competitive ability in the two different bedbug lineages. The proportion of sired offspring (paternity; P2) when tester females were mated first with males from the same population and subsequently with focal males differing in diet (original or foreign, resp. bat or human blood) and genetic origin (bat (BL, purple colour) or human lineage (HL, green colour)). (**a**) The change of P2 over the egg laying period varied between lineages and diets: P2 in HLs increased at a slower rate on human than on bat blood. Lightly coloured lines represent the average P2 over egg laying for each population and the dark coloured lines with 95% confidence intervals show the average P2 for the lineages. Solid lines correspond to the original diet, dashed lines to foreign diet. (**b**) The male's origin or diet showed no clear effect on the overall P2, even though P2 seems to be generally higher on bat blood. Grey symbols represent individual data points. Coloured symbols and error bars show the mean and standard error for the individual male population (different colour shades and point shapes).

Focal males exhibited an increase in paternity (P2) share over time that differed for lineages and diets, thanks to significant week x lineage and nearly significant week x diet interactions (Table 2). The differences in the increase were more noticeable for HL males than BLs because HL males reached long-time about 50% paternity on original diet but close to 100% on foreign diet, which was similar to P2 of BL males (Fig. 2a). While for BLs, there was no clear difference between diets and we can just speculate if BLs are overall genotypically better than HLs and thus compensate the effect of worse diet. Nevertheless, the total P2 did not significantly differ with respect to male diet, lineage, or their interaction (Table 2, Fig. 2b).

**Table 2:** Statistical results for GLME analysis of focal male paternity (P2) over time (n = 440 mother-week combination) and in total (n = 69 families) when female was mated with male from same population at first and after with focal male. Values in bold represent significant effects and nearly significant effects are underlined.

	P2 over time				Total P2	
	χ^2^	*p*	Post-hoc tests		χ^2^	*p*
			z	*p*		
Week	87.92	** < 0.001**	9.38	** < 0.001**	–	–
Lineage	3.21	0.073	1.79	0.073	1.48	0.224
Diet	0.44	0.505	0.67	0.505	1.12	0.291
Week : Lineage	15.61	** < 0.001**	–3.95	** < 0.001**	–	–
Week : Diet	3.79	0.052	−1.95	0.052	–	–
Lineage : Diet	–	–	–	–	2.64	0.104

## Discussion

Our data reveal a relatively strong environmental effect on male competitive fertilisation success, which, we believe, has important consequences for the theory of sperm competition. However, we found that the effect of male diet varies with the male's genetic origin, leading us to reject the idea that local adaptation to the environment caused the genetic effect. By contrast, the overall beneficial effect of bat blood reveals phenotypic plasticity in sperm performance in response to diet. As a consequence, an asymmetric genetic homogenisation of populations would be expected. Therefore, the genomic separation between bat- and human-associated bedbugs (*C. lectularius*)^41,42^, seems not driven by male fertility and sperm competition. Therefore, the separation must even be stronger than previously thought to maintain genetic separation in the face of the predicted homogenising effect of dietary impacts on sperm performance.

### Dietary effects on sperm competition

In polyandrous species, sperm competitive ability is arguably the most important male fitness trait. It is considered related to the genetic quality of a male and therefore evolving^59,60^ and consequently a predictor of evolutionary change. However, other studies (reviewed by Dobler and Reinhardt 2016^8^) have shown that the current empirical evidence is not consistent with the idea that sperm competition is generally heritable. Instead, it has a strong environmental component. The generally large effect of the environment on sperm function^7^ suggests that sperm competition studies should not neglect environmental effects. Here, despite the non-significant diet effect, we observed paternity increase, on average, between 5 and 10% higher on bat blood than human blood for all populations regardless of lineage. We also found that paternity in HL males increased over time at a slower rate on human than on bat blood. Both results represent clear evidence that the environment shapes the outcome of sperm competition. These results are in contrast with a study on *Drosophila* that found no effect of macronutrient intake on either sperm defence and offence^61^ but support previous examples showing environmental effects on sperm competitive ability in fish^62^, cricket species^4^ and *Drosophila melanogaster*^8^. The latter study explicitly followed the time course of P2, as we did herein. This study as well as our current one, both found little overall variation in P2 but in its temporal changes. Methodologically, this suggests that studies following the temporal change in P2 may have higher resolution and that the overall P2 result will depend on the time over which P2 is sampled. Biologically, the temporal variation in P2 means that the timing of dispersal in relation to the timing of mating affects patterns of gene flow.

An interesting question pertains to the mechanistic reason of why bat blood is superior for sperm competition than human blood. Bat blood is very different from the blood of other mammals, including humans^63^. For example, bat erythrocytes are smaller and more numerous than human erythrocytes^64^. If erythrocytes, rather than plasma, form a major part of the bedbugs' diet, then variation in nutrients or other characteristics between the two diets may impact sperm production or sperm performance. For example, variation in protein and carbohydrate intake influences sperm production and translates into male fertility in cockroaches^65^, in Queensland fruit flies^66^ and in ants^67^. In humans and *Drosophila*, dietary fats also play a role in defining sperm quality and quantity^27,68^.

Mechanistically, the paternity change from 30% towards nearly 100% (Fig. 2a) suggests that the pattern we found is consistent with the so-called passive sperm loss model (e.g. Birkhead et al. 1999^69^; Colegrave et al. 1995^70^; Simmons 2001^71^; Tsubaki and Yamagishi 1991^72^; Yamagishi et al. 1992^73^) shows that. In this model, sperm of both males mix in the female's storage organ, but sperm from the first male is slowly disappearing from the female's storage site. Interestingly, it was argued later that such a pattern is not only observed when assuming an exponential loss of sperm number but also when assuming an exponential loss of sperm quality^74^. As such, it is not clear whether HL males reared on human blood only reached 50% paternity because females would—somehow—prevent the loss of the first male's sperm or because the sperm quality would simply not reach that seen in other treatments. Future studies should look into how differences between bat and human blood translate into differences in sperm composition.

Our HL populations were in the laboratory for a longer time than the BL populations and because we used an HL population as a tester line, local adaptation and genetic match would predict that BL populations do worse and show higher variance than HL populations. However, neither was the case, suggesting that the effects we observed are not artefacts of our laboratory breeding. Particularly striking was the fact that HL males did worse on human blood than on bat blood.

### The role of environmental effects on sperm function in speciation

Gene flow and environmental factors predict local adaptation, i.e. in our system we had expected that HL males had better sperm performance on human blood, BL better sperm performance on bat blood. We firmly reject this prediction here because our data clearly show that bat blood generally improved sperm performance of HL males. In terms of temporal variation in paternity, we found the same pattern but did not find that BL males performed worse on human blood. In other words, we see maladaptation in the sperm performance of HL males. Albeit maladaptation, or perhaps cost of adaptations to new or extreme environments, is common, at least in animal breeding^75^. These effects usually show up only for a short time. By contrast, the BL and HL lineage separated tens or hundreds of thousands of years ago^41,42^. Our results answer a key question in speciation, the relative role of genetic and ecological divergence in reproductive isolation at the post-mating level; and they do so in a massively re-emerging human parasite.

## Data availability

All relevant data are within the paper as Supporting Information files (Tables S1–3) and in dataset repository Figshare https://doi.org/10.6084/m9.figshare.12046896.

## Supplementary Information

Supplementary Information.
