# Supplementary material for: Male diet affects female fitness and sperm competition in human- and bat-associated lineages of the common bedbug, Cimex lectularius
Source: Sci Rep. 2021 Jul 30;11:15538. doi: 10.1038/s41598-021-94622-6 (PMC8324850; doi:10.1038/s41598-021-94622-6)
Supplement: Supplementary file 1 — Supplementary Information. [file 41598_2021_94622_MOESM1_ESM.docx]

SUPPLEMENTARY INFORMATION FOR

**Male diet affects female fitness and sperm competition in human- and bat-associated lineages of the common bedbug, *Cimex lectularius***

**INDEX:**

Supplementary tables

Table S1 – Samples sizes for both experiments on the effect of focal male diet

Table S2 – Microsatellite primers

Table S3 – PCR protocol

**Table S1** Sample sizes for both the effect of male diet on female fecundity (Exp. 1) and on sperm competition (Exp. 2) and the number of families usable for the paternity (Paternity) analysis separated for males from bat- (BL) and human-related populations (HL).

| **Population** | **Diet** | **Blood** | **Exp. 1** | **Exp. 2** | **Paternity** |
| --- | --- | --- | --- | --- | --- |
| *BL* |  |  |  |  |  |
| BL1 | Original | Bat | 14 | 11 | 11 |
| BL1 | Foreign | Human | 7 | 6 | 5 |
| BL2 | Original | Bat | 12 | 12 | 12 |
| BL2 | Foreign | Human | 12 | 8 | 6 |
| *HL* |  |  |  |  |  |
| HL1 | Original | Human | 14 | 10 | 9 |
| HL1 | Foreign | Bat | 11 | 11 | 11 |
| HL2 | Original | Human | 11 | 10 | 6 |
| HL2 | Foreign | Bat | 11 | 11 | 9 |

**Table S2** Description of primers arranged in 2 multiplex mixes. T_a_ = annealing temperature (^o^C) and total number of observed alleles across all parents (N_A_).

| **Locus** | **Sequence (5’ – 3’)** | **Multiplex** | **T_a_** | **Expected/**  **Observed size (bp)** | **N_A_** |
| --- | --- | --- | --- | --- | --- |
| 47A04 | F: CCATTGACGGAGGGTTGCTTC | 1 | 60 | 164 | 7 |
|  | R: CACTTTCTTGTAACCATCACCATC |  |  | 166 - 217 |  |
| BB454_20 | F: GCAACCCTGGACTTCTCAAC | 1 | 60 | 188 | 5 |
|  | R: TCAGCTCTCCATTAGAACGAAAC |  |  | 235 - 263 |  |
| 48G02 | F: TCATATGGGCGGATTAGAGC | 1 | 60 | 302 | 11 |
|  | R: TAACAATCTGGAGGCGGAAC |  |  | 291 - 350 |  |
| 46H08 | F: TTGTGAGTGTGTCTCTCTCTACTGTG | 2 | 60 | 144 | 3 |
|  | R: CAGGTTCACAGGCCAAATG |  |  | 140 - 163 |  |
| 48E11 | F: TTCGTTTGTGTAGAACCTTGG | 2 | 60 | 269 | 7 |
|  | R: TACGTCCCTACAAGCTCACC |  |  | 217 - 281 |  |
| 47A07 | F: AGATAGGGCAACCTTTCAGAG | 2 | 60 | 315 | 3 |
|  | R: TTGGTGATAGTGAACGAACG |  |  | 309 - 353 |  |

**Table S3** PCR protocol

| **Step** | **Temperature (°C)** | **Duration (min)** | **Cycles** |
| --- | --- | --- | --- |
| Initial Denaturation | 95 | 15:00 | 1 |
| Subsequent Denaturation | 94 | 0:30 |  |
| Annealing | 58 | 1:30 | 30 |
| Extension | 72 | 01:00 |  |
| Final Elongation | 60 | 30:00 | 1 |
